# Criminal Justice Involvement after Release from Prison following Exposure to Community Mental Health Services among People Who Use Illicit Drugs and Have Mental Illness: a Systematic Review

**DOI:** 10.1007/s11524-022-00635-5

**Published:** 2022-05-02

**Authors:** Ashleigh C. Stewart, Reece D. Cossar, Brendan Quinn, Paul Dietze, Lorena Romero, Anna L. Wilkinson, Mark Stoové

**Affiliations:** 1 Behaviours and Health Risks, Burnet Institute, 85 Commercial Rd, Melbourne, 3004 Australia; 2 School of Public Health and Preventive Medicine, Monash University, Melbourne, Australia; 3 Centre for Forensic Behavioural Science, Swinburne University of Technology, Melbourne, Australia; 4 National Drug Research Institute, Curtin University, Melbourne, Victoria Australia; 5 Ian Potter Library, Alfred Health, Melbourne, Australia

**Keywords:** Mental health, Prison, Systematic review, Illicit drug use, Criminal justice involvement, Community mental health services

## Abstract

Illicit drug use and mental illness are common among people in prison and are associated with higher rates of reoffending and reimprisonment. We conducted a systematic review, searching MEDLINE, Embase, and PsycINFO to January 10, 2022, for studies reporting criminal justice involvement following exposure to community mental health services among people released from jail or prison who use illicit drugs and have mental illness. Our search identified 6954 studies; 13 were eligible for inclusion in this review. Studies were separated into three broad categories based on community mental health service type. Eleven of 13 studies reported a reduction in criminal justice involvement among participants exposed to community mental health services compared to a comparison group. Findings indicate a need to expand and improve integration and referral mechanisms linking people to community mental health services after jail or prison release, alongside a need for tailored programs for individuals with complex illicit drug use and mental health morbidities.

**Supplementary Information:**

The online version contains supplementary material available at 10.1007/s11524-022-00635-5.

## Background

People involved in the criminal justice system experience high rates of health, social, and economic inequality [1–7]. Substance use disorder (SUD) is common among people involved in the criminal justice system (global estimates indicate 30% of males and 51% of females in prison have SUD [8]) and is associated with poor health and social outcomes, including elevated risk of infectious disease [9–11], unstable housing [12, 13], and mortality [14–17]. Illicit drug use after prison release is also associated with increased risk of reimprisonment [18, 19].

Mental illness is also common among people in prison [6, 20–22]. A recent systematic review reported one in seven people in prison globally is diagnosed with a serious mental illness, such as psychosis or major depression [6]. Estimates of co-occurring SUD and mental illness among people in prison range from 21 to 29% [5, 23], and this co-morbidity is associated with even greater incidence of poor health and social outcomes after release from prison compared to people in prison without co-occurring illness including hospital presentations [23], arrest, being charged with offences [24], and reimprisonment [25].

The deinstitutionalisation of people with mental illness and the decommissioning of large mental health facilities internationally have resulted in many positive outcomes for people with mental illness as a result of health care and social support being provided in community settings [26–28]. However, the shift towards community-based mental health services, coupled with resource constraints and inadequate service provision [26], has also resulted in individuals with the highest need, such as those with serious mental health illness (e.g., psychosis and personality disorders) or co-morbidity, seeking care within an overburdened community mental health and non-mental health system that is often ill-equipped to meet these needs [29]. The circumstances in which people are released from prison, such as into unstable housing [2, 30], present additional challenges in maintaining engagement with mental health care, and co-occurring illicit drug use can add further complexity to service provision. Community mental health services are now typically the largest component of the mental health care system, particularly within high income countries [31], and range from low- to high-level care with the aim to support and enable people with mental illness to live independently within the community. Given the high prevalence of mental illness [6] and co-occurring SUD and mental illness [5, 23] among prison populations, alongside social and economic marginalization upon release, standard community mental health services may not be adequate to meet the needs of this population when transitioning from prison to the community.

Despite presumed benefits accruing from mental health service contact after release from prison, there has been no systematic review of the effect of accessing community mental health services on future criminal justice involvement among people who use illicit drugs and people who use illicit drugs and have mental illness after they are released from prison. Considering evidence of the compounding effects of illicit drug use and mental illness on poor post-release outcomes and the significant social and economic burden associated with reoffending in this group, we present a systematic review of the published literature exploring whether community mental health service contact helps prevent recidivism and reimprisonment among people who use illicit drugs and have mental illness.

## Methods

### Search Strategy

This systematic review of peer-reviewed literature was conducted in accordance with the Preferred Reporting Items for Systematic Reviews and Meta-Analyses (PRISMA; Appendix 1 in Supplementary material). The methods used were consistent with previous systematic reviews of people involved in the criminal justice setting [3, 23] and people who use drugs [32]. The Joanna Briggs Institute (JBI) Checklist for Cohort Studies [33] was used for the critical appraisal of each study included (Appendix 3 in Supplementary material). The protocol was registered on PROSPERO (number CRD42020207629).

We searched electronic peer-reviewed literature databases (MEDLINE [Ovid], Embase [Ovid], and APA PsycINFO [Ovid]) using indexed terms, subject headings, and free text words, developed in consultation with a specialist systematic review librarian (LR; Appendix 2 in Supplementary material). Searches were performed from database inception to January 10^th^, 2022. Search results were not limited by date but were limited to English language articles. We included comparison studies that reported on involvement with the criminal justice system (i.e., recidivism, re-arrest, reconviction, and reimprisonment) following exposure to a community mental health service(s) among people who use illicit drugs and people who use illicit drugs and have mental illness. For the purpose of this study, diagnosis of SUD was not considered a mental health issue. We included a broad range of subject headings (e.g., MeSH terms and EMTREE subject headings) and key words that included “criminal justice”, “illicit drug use”, “mental illness”, and “community mental health care” to capture studies of people who use drugs who may have had either diagnosed or undiagnosed mental illness who come into contact with community mental health services after release from correctional facilities. We did not distinguish between people released from jail, which typically refers to people on remand who are awaiting sentencing, and people released from prison, which typically refers to people who are sentenced.

### Extraction and Screening

Citations were exported from respective databases and imported into an Endnote (version X9) library to identify and remove duplicates. The Endnote library was imported into Covidence, and any further duplicates removed. Screening of title and abstract, followed by full-text review, was done by two reviewers (AS and RC). Studies were included following full-text review if they met the inclusion criteria below:

- Involved participants released from correctional facilities (jail or prison); and
- Involved people who use(d) illicit drugs (by any route of administration); or
- Involved people who use illicit drugs and have mental illness; and
- Reported contact with community mental health services following previous contact with the criminal justice system;
- Reported estimates of involvement with the criminal justice system following exposure to community mental health services;
- Included a comparison group (e.g., within cohort comparison or other control group); and
- Was a peer-reviewed study; and
- Written in English.

Full-text review was independently performed by two reviewers (AS and RC). A third reviewer (MS) was available to resolve discrepancies if consensus was not reached (consensus was reached in all instances). Data from eligible studies were extracted into a purpose-built Microsoft Excel table. Data were then checked for accuracy against the original source by two authors (AS and RC). All extracted data were categorised by country. From eligible studies, we extracted data on the study year, follow-up time, study design and sample size, sample description, illicit drug use, community mental health service exposure/control measure and data type, and criminal justice involvement outcome measure and data type.

### Measures

Criminal justice outcomes measured after contact with community mental health services included recidivism, re-arrest, reconviction, and reimprisonment. Based on the type of mental health care or intervention provided, community mental health service exposures assessed in the included studies were categorised into three mutually exclusive groups: (1) standard community mental health service contact, (2) tailored prison transitional support programs and case management, and (3) specific effect of exposure to antipsychotic and mood stabilizing medication management.

## Results

The search yielded 8872 studies, with 6954 eligible for screening after duplicates were removed. Screening of titles and abstracts excluded 6875 studies and a further 66 were excluded following full-text assessment, resulting in 13 studies included for analysis in this systematic review (flowchart accessible in Appendix 4 in Supplementary material). Characteristics of the included studies are summarised in Table 1. Ten studies were from the Unites States of America (USA) [34–44], one from Canada [42], one from the UK [45], and one from Australia [46]. Studies were conducted between 1983 and 2016. Illicit drug use among study participants varied in definition (e.g., SUD diagnosis or self-reported illicit drug use) and in its consideration in analyses by research design (e.g., within study eligibility criteria or included as a covariate within multivariable analysis). All studies included participants who both used illicit drugs and were identified as having a co-occurring mental illness. Hereafter, the population of interest will be referred as “people who use illicit drugs and have mental illness”.

**Table 1:** Study-level information for estimates of subsequent contact with the criminal justice system after community mental health service intervention among people who use drugs

	Country	Study year	Follow-up time	Study design and sample size (*N*)	Sample description	Illicit drug use	Community mental health service exposure/control	Exposure measure data type	Criminal justice outcome measure	Outcome measure data type
Constantine et al (2012) [34]	USA	2002–2007	27–55 months	Retrospective cohort; *N*=11524 (*n*=3769, Florida; *n*=7755, Texas)	All adults with SMI who had contact with mental health services and who were detained in County jail during the observation period	86% in the Florida cohort and 79% in the Texas cohort had SUD.	Standard community mental health inpatient/ED contact and outpatient contact (linked data).	Linked data to statewide health and social service datasets including inpatient and outpatient claims, and involuntary admissions.	The risk of arrest after controlling for demographic and diagnostic characteristics.	Linked data to county criminal justice information systems containing information about all individuals in contact with criminal justice.
Domino et al (2019) [35]	USA	2006–2007	12 months	Retrospective cohort; *N*=3086 (*n*=871, referral group; *n*=2215, non-referral group)	All adults with severe mental illness who were released from prison during the first two years of an expedited Medicaid policy	57% in the referral group and 53% in the non-referral group had alcohol or drug use disorder.	Medicaid funded inpatient and outpatient mental health services vs. standard public mental health services (linked data).	Linked data including measures of inpatient and outpatient mental health service use from Medicaid claims and public mental health system utilization.	Probability of criminal justice involvement (re-arrest, reimprisonment) in the year following index release.	Linked data to Department of Corrections and state patrol records to determine criminal justice system involvement during 12 months post–index release: arrests, jail use, and any imprisonment.
Farabee & Shen (2004) [36]	USA	n.d	12 months	Prospective non-randomized cohort; *N*=200	Parolees referred to a community-based psychiatric outpatient clinic, enrolled within 30 days and prescribed antipsychotics <1 week prior to enrollment	60% of participants who provided hair samples (*n*=140) tested positive for an illicit substance.	Antipsychotic and mood stabilizing medication adherence.	Medication adherence was determined via self-report data and urine samples tested for medication trace levels.	The odds of reimprisonment after controlling for substance use, age, gender, and ethnicity.	Linked data to the offender-based information system to determine reimprisonment for either a new charge or for violation of the terms of parole within the 12 months following release from prison.
Godley et al (2000) [37]	USA	n.d	6 months	Prospective non-randomized cohort; *N*=54	Adults with major psychiatric diagnosis defined in the DSM-IV and a co-occurring diagnosis of substance dependence who come in contact with County jails	All participants had SUD classified by DSM-IV.	Referral and case management via intervention—Treatment Alternatives for Community Safety.	Data collected via the case management intervention on program engagement.	Past six month police contacts, imprisonments, and number of days spent in prison was measured at intake (baseline) and at six month follow-up.	Data source not clearly stated.
Green et al (2016) [46]	Australia	2010–2014	4 years	Prospective non-randomized cohort; *N*=63	Adults in correctional centres who were referred to the Transitional coordination program (TCP)	70% in TR-long (≥62 days of support), 77% in TR-short (<62 days of support), and 60% in TCP only had a lifetime history of SUD.	CMHS via single or combined TCP and TRRanS programs (linked data).	Mental health hospitalization and treatment-related data were obtained from the state-based Health Consumer Integrated Mental Health Application database.	Time-to-reimprisonment and factors associated with reimprisonment after controlling for age, SUD, diagnosis of psychosis, and prior imprisonments.	Linked data from statewide Corrective Services Integrated Offender Management System to determine reimprisonment episodes.
Hall et al (2012) [38]	USA	2006–2007	683–1410 days (median 1044 days)	Prospective cohort; *N*=2005	All adults identified with SMI transitioning from prison to the community setting in New York State	60% had a diagnosis of SUD.	Standard CMHS and tailored PSTP	Linked data to the Mental Health Automated Record System of inpatient admissions before and after prison release and outpatient contacts after prison release, Medicaid-reimbursed mental health-related clinic and hospital visits following prison release, and data on transitional mental health service contacts.	Factors associated with time to re-arrest following index prison release.	State-based Computerized Criminal History file provided criminal history data and all imprisonments and releases for the purpose of measuring time at risk after release.
Hawthorne et al (2012) [39]	USA	2004–2007	12 months	Retrospective cohort; *N*=39,463	All patients identified with mental illness who received ≥1 CMHS and had a matched record with the San Diego County jail	65% of participants reimprisoned during the study period had co-occurring mental illness and SUD.	Standard CMHS, including outpatient and case management services.	Linked data to adult mental health services system of all adult psychiatric patients who received at least one mental health service contact during the study period.	Factors associated with risk of reimprisonment within one year of index prison release.	Linked data to the Sheriff’s Department Jail Information Management System of incarceration records during the study period.
Kesten et al (2012) [40]	USA	1998–2004	6 months	Retrospective cohort; *N*=971 (*n*=883, DMHAS; *n*=88, CORP). Sample randomization not stated.	Adults with mental illness leaving prison in Connecticut	All participants in CORP program and 66% in DMHAS had co-occurring mental illness and SUD.	Standard CMHS vs. specialised reentry program including tailored community services engagement.	Linked data to the CORP and DMHAS.	Rates of re-arrest between participants in the CORP and those receiving standard CMHH.	Linked data from the Department of Corrections of re-arrests within 6 months following release from prison.
Lovell et al (2002) [41]	USA	1996–2000	27–55 months (mean=39 months)	Retrospective cohort; *N*=5304 (*n*=337 people with mental illness; *n*=4967, comparison group)	All identified people with mental illness released from Washing State prison in 1996–1997.	49% recorded substance abuse.	Standard CMHS (linked data).	Linked data to the Department of Corrections to determine mental illness diagnoses and data from the mental health division of community mental health contacts.	Recidivism associated with timing and frequency of exposure to mental health services.	Linked data from the state-based Department of Corrections of recidivism following release from prison.
Sahota et al (2009) [45]	UK	1983–2003	Mean 10 years	Retrospective cohort; *N*=163 (*n*=70 participants discharged to community forensic services; *n*=93 discharged to general services).	All patients admitted and discharged from Arnold Lodge medium secure unit during the period of follow-up.	16% reported use of alcohol and drugs within one year post prison release.	Specialist community forensic service vs. general service	Administrative data from county-based mental health databases, discharge summaries, clinic letters, and legal reports regarding CMHS type.	Time spent in the community prior to reconviction following discharge from a medium secure unit.	Administrative data from the Offenders Index, the Police National Computer, and the Home Office Mental Health Unit to determine reconvictions.
Stewart et al (2017) [42]	Canada	2007–2008	Recidivism= 3–6-month follow-up. Reimprisonment= 24–48-month follow-up.	Retrospective non-randomized cohort; *N*=646 (CMHI patients receiving clinical discharge planning only, *n*=65; CMHI patients receiving CMHS services only, *n*=249; CMHI patients receiving CDP and CMHS services, *n*=63; and untreated patients who would have met eligibility for CMHI, *n*=269).	Men leaving prison with SMI in Canada.	81–89% identified with co-occurring mental illness and SUD.	CMHI including CDP and/or CMHS vs. none.	Data come from the CMHI cohort to identify people who participated in the treatment and comparison groups.	Rates of reoffending and reimprisonment following release from prison, and time-to-reoffending and reimprisonment after controlling for demographic and clinical factors between the intervention and no-intervention groups during a fixed follow-up period.	Linked data from the offender management system of profile and case management information and the police information database for data on criminal recidivism.
Vigilante et al (1999) [43]	USA	1992–1995	3 and 12 months	Retrospective non-randomized cohort; *N*=430 (*n*=78, WHPPP; *n*=352, control group).	Women with HIV in adult corrective services at risk of reimprisonment.	88% reported drug use, of which 77% reported IDU.	Pre-release discharge planning and post-release case management, incl., substance use treatment and CMHS (linked data).	Data on women in the WHPPP were collected from chart review, prison databases, and a participant questionnaire.	Differences in rates of recidivism following index prison release were compared between the WHPPP and control groups.	Linked data from the statewide prison database was used to determine recidivism rates at three and 12 months following release from index imprisonment.
Wang et al (2019) [44]	USA	2013–2016	12 months	Retrospective non-randomized cohort; *N*=188 (*n*=94, TCN patients; *n*=94 matched controls).	Adults released from Connecticut Department of Correction prison system who received primary care between May 2013 to February 2016 and who had a chronic health issue (including mental health).	35% of TCN patients had opioid abuse/dependence (39% for controls).	Primary health care support and assisted referrals to CMHS via the TCN program.	TCN program data and linked data from the Mental Health and Addictions Services, Department of Social Services, and Department of Public Health to determine patient characteristics and subsequent CMHS contacts.	The odds of reimprisonment and the number of days spent in prison between TCN participants and controls within 12 months of index prison release.	Linked data from the Department of Corrections and Court Support Services Division databases to determine reimprisonment within 12 months of index prison release.

Note: *USA*, United States of America; *UK*, United Kingdom; *DMHAS*, Department of Mental Health and Addictions Services; *CORP*, Connecticut Offender Re-entry Program; *TCN*, Transitions Clinic Network; *WHPPP*, Women’s HIV Prison Prevention Program; *CMHI*, Community Mental Health Initiative; *CDP*, Clinical Discharge Planning; *TCP*, Transitional Coordination Program; *TRRanS*, Transition Reintegration Recovery and Support; *TR-long*, Combined TRRanS and TCP support for ≥62 days; *TR-short*, Combined TRRanS and TCP support for <62 days; *HIV*, human immunodeficiency virus; *CMHS*, Community Mental Health Services; *SUD*, substance use disorder; *DSM-IV*, Diagnostic and Statistical Manual of Mental Disorders, 5th Edition; *SMI*, serious mental illness (e.g., psychosis and major depressive disorders); *IDU*, injecting drug use

Of the 13 studies included, 12 used linked administrative data from local corrections and criminal justice databases (e.g., police records, court records, and prison data) to determine the primary outcome measure (i.e., criminal justice involvement after exposure to community mental health services). One study did not report clearly the source of their outcome data [37]. Specific criminal justice outcomes varied. Six studies reported reimprisonment [36, 37, 39, 44, 46], three studies reported re-arrest [34, 38, 40], two studies reported recidivism [41, 43], one study reported reconviction [45], and two studies reported both re-arrest and reimprisonment [35, 42]. Measurement of primary outcomes also varied, including analysing associations of criminal justice involvement following exposure to community mental health services after controlling for covariates [34, 35, 38, 39, 44], measuring rates of criminal justice involvement [40, 42, 43], reporting time-to-event of criminal justice involvement [42, 45, 46], measuring the timing and frequency of criminal justice involvement following exposure to community mental health services [41], and the number of days spent in prison following exposure to community mental health services (Table 1) [44].

Eleven studies used linked administrative data from local mental health service databases, or administrative data specific to a tailored mental health intervention to measure community mental health service exposures. Community mental health services included standard mental health care available in the community (i.e., provided through pre-existing general mental health services) [34, 35, 38, 39, 41], tailored prison mental health transitional support programs and case management [37, 39, 40, 42–46], and for one study, antipsychotic and mood stabilizing medication management, which measured exposure using a combination of self-report and biological samples (e.g., urine samples; Table 1) [36].

Community mental health service exposures in eight studies were exclusively mental health care-focused [34–36, 38, 39, 41, 42, 45], and five integrated additional support, including for physical health, alcohol and other drug use, and social and vocational assistance [37, 40, 43, 44, 46]. Most mental health interventions were described as involving a multidisciplinary team, including clinical health care providers, community support staff, and peer supports (Table 1).

The following section presents a narrative synthesis of criminal justice involvement following exposure to community mental health services and is presented by type of mental health service exposure: (1) standard community mental health services operating within pre-existing mental health service structures, (2) tailored prison transitional support programs and case management, and (3) mental health medication management.

### Standard Community Mental Health Care Contact

‘Standard community mental health services’ in this review refers to established standard or general community mental health services within a pre-existing service structure and was assessed in five of 13 included studies. Two studies described criminal justice involvement within a period following last community mental health service contact [34, 38]. Hall et al. [38] reported exposure to community mental health services resulted in a 16% reduction (*p=*0·036) in the odds of re-arrest in the month following last service contact. Constantine et al. [34] found a greater reduction in the odds of re-arrest closer to the receipt of last community mental health service contact (measured three monthly) compared to those without a community mental health contact, with the odds of re-arrest reduced by 17% (*p*<0·001) within 90 days, by 11% within 180 days (*p*<0·001), and by 9% within 270 days of last mental health contact. Conversely, odds of re-arrest increased by 23% (*p*<0·001), 80% (*p*=0·01), and 11% (*p*=0·01) within 90, 180, and 270 days of last contact with emergency/inpatient mental health services, respectively [34].

Lovell et al. [41] described the timing of criminal justice involvement following exposure to community mental health services. Participants received an average of 3 months of community mental health services in the first year post-release from prison [41]. They found participants involved in new criminal offenses received community mental health services on average 2 months later and had on average 40% fewer monthly service contact-hours than people who were not involved in new criminal offenses. While these differences were substantial, only 135 offences were recorded across the study and the difference was not statistically significant. Hawthorne et al. [39] examined factors associated with reimprisonment and found standard community mental health service contact was associated with a reduction in the risk of reimprisonment. The median time between release and receiving a community mental health service contact was 17 days [39]. After controlling for sociodemographics, mental illness diagnostic category, and baseline imprisonment characteristics, they estimated that participants who had community mental health service contacts reduced their odds of reimprisonment within 1 year of release from prison by 36% (*p*<0·001) compared to those without mental health service contact.

Finally, one study examined the impact of mental health service contacts (via the provision of expedited Medicaid referral within 31 days of release from prison) on criminal justice involvement (e.g., arrest, jail, or prison) within 12 months following release from prison [35]. Domino et al. [35] found that among those receiving expedited referral, 30% received both a mental health service contact and a psychiatric medication prescription, compared to only 6% of those without expedited referral. They found those receiving expedited services had greater odds of reimprisonment, which was primarily driven by high rates of violations of conditions of release (e.g., failure to comply with mental health treatment plans or to attend meetings with probation officers, or positive alcohol and drug urine analysis) within 12 months of release from prison [35]. This may suggest mental health treatment was functioning as a form of monitoring, and therefore increasing technical violations. However, participants with expedited Medicaid enrolment had a higher criminogenic risk profile (e.g., higher prevalence of alcohol and drug disorder, a longer sentence length, and more serious criminal convictions for their index imprisonment) compared to participants without expedited Medicaid enrolment [35].

### Tailored Prison Transitional Support Programs and Case Management

Tailored﻿ prison transitional support programs and case management’ were defined in this review as programs not otherwise routinely provided in community mental health services that offered post-release mental health treatment and support tailored to people transitioning into the community from prison. Eight studies reported on these tailored programs.

Three studies reported reductions in criminal justice involvement after exposure to a tailored mental health intervention compared to a control group [40, 43, 46]. Vigilante et al. [43] reported recidivism outcomes among a non-randomised cohort of women with HIV who participated in a Women’s HIV Prison Prevention Program (WHPPP) that included pre-release planning and post-release support (e.g., mental health services, housing support, financial aid) from a multidisciplinary team [43]. WHIPP recipients demonstrated a 4% and 12% reduction in recidivism compared to no-intervention controls at 3 and 12 months following release from prison, respectively [43]. Seventeen percent of WHPPP recipients returned to prison compared to 41% of those not in the program. Of the women who were reimprisoned, those in the WHPPP spent more time in the community prior to returning to prison, compared to women not in the program [43]. Kesten et al. [40] assessed re-arrest within 6 months following release from prison among participants with mental illness enrolled in the Connecticut Offender Re-entry Program (CORP), which involved mental health treatment and group counseling sessions (fortnightly sessions conducted over 9–12 months) facilitated by a psychologist, compared to participants receiving standard treatment planning services from a state-based mental health agency. All participants enrolled in the CORP had co-occurring SUD and mental illness, compared to only 66% of participants receiving standard treatment and planning. Despite this, 14% of participants in the CORP were re-arrested within 6 months of prison release compared to 28% who were re-arrested in the standard treatment and planning group [40]. Green et al. [46] estimated the effect of referral to a Queensland Prison Mental Health Transition Coordination Program involving pre-release discharge planning and/or the provision of post-release transitional support on time to reimprisonment. After controlling for age, SUD, diagnosis of psychosis, and number of prior episodes of imprisonments, they found every additional month of transitional support was associated with a 14% reduction in the risk of reimprisonment compared to participants receiving pre-release discharge planning only [46]. The study also found participants receiving a longer duration of support (e.g., more than 2 months) spent more time in the community prior to reimprisonment [46].

Stewart et al. [42] analysed rates of returning to reoffending and reimprisonment following release from prison after controlling for time in the community post-release (“time at-risk”) and other clinical (e.g., program intervention status and substance dependence) and offence-related (e.g., sentence number and violent offence) covariates. Participants were men diagnosed with serious mental illness (i.e., psychosis, major depression, or personality disorder) and received one of three increasingly comprehensive post-release interventions (pre-release clinical discharge planning only, tailored post-release community mental health intervention only, or both) and were compared to a control group of men who were eligible but did not receive the intervention [42]. Participants receiving the post-release community mental health intervention demonstrated lower rates of return to custody at 3-month (2% vs. 8%, *p*<0·05) and 6-month (16% vs. 30%, *p*<0·05) follow-ups, and lower rates of recidivism at 24-month (30% vs. 51%, *p*<0·05) and 48-month (36% vs. 61%, *p*<0·05) follow-ups, compared to the control group [42]. Participants receiving the post-release intervention also demonstrated lower rates of return to custody and recidivism compared to those receiving pre-release clinical discharge planning only and those who received both interventions. After controlling for time-at-risk and other covariates, compared to the control group, participants receiving the post-release intervention alone had a 42% lower risk of reoffending (*p*<0·001) and a 49% lower risk of reimprisonment (*p*<0·001). Participants who received the combined pre-release and post-release interventions had a 28% lower risk of reoffending and 30% lower risk of reimprisonment compared to the control group; however, these results were not statistically significant [42].

As part of a program evaluation, Godley et al. [37] analysed differences in criminal justice involvement among participants waiting for sentencing who were diverted to probation and case management via the Treatment Alternatives for Safe Communities program for people with co-occurring mental illness and substance abuse problems (determined by the DSM-IV). Program recipients were assigned two case managers who facilitated access and referral to substance use treatment and other services and social supports, and on average meet with clients weekly (approximately 10 clients to one case manager) [37]. Evaluation data was collected at program intake (i.e., upon diversion) and approximately 6 months following intake. The prevalence of past 6-month legal problems decreased from 95% at intake to 70% at 6-month follow-up (*p*=0·006) among program recipients, and the percentage of recipients who were jailed decreased from 74% at intake to 26% at 6-month follow-up (*p*=0·000) [37]. Additionally, the number of days spent in jail in the past month decreased from 7 days at intake to 2 days at follow-up (*p*=0·012) and days spent in jail in the past 6 months decreased from 39 days at intake to 8 days at follow-up (*p*=0·005) [37].

The study by Hall et al. [38] explored factors associated with re-arrest among participants leaving prison with serious mental illness, including exposure to a Parole Supported Treatment Program (PSTP) that involved mental health case management, treatment, psychiatric consultation, and supported housing (PSTP eligibility was restricted to those experiencing both SUD and serious mental illness) [38]. Case managers designated through the PSTP were assigned reduced caseloads (approximately 25 clients to one case manager) and regularly met with parolees [38]. Among 60 participants exposed to the PSTP intervention, after controlling for sociodemographic and crime factors, there was a 46% reduction (*p*=0·006) in the risk of re-arrest after release from prison during a mean follow-up period of 1045 days [38]. However, assignment to the PSTP was not randomised, which may have affected the outcome.

Wang et al. [44] described the number of days spent in prison among participants reimprisoned, who also experienced chronic health conditions, including mental illness, and were enrolled in the Transitions Clinic Network (TCN) program. The TCN program involved an interdisciplinary team, including community health workers with a history of imprisonment, who supported people leaving prison via referrals to primary health care, and where appropriate, to mental health and community (e.g., housing and food access) services [44]. Participants enrolled in the TCN program spent 45% fewer days reimprisoned within 12 months of their prison release date, compared to a matched comparison group that were not enrolled in the TCN program [44].

Sahota et al. [45] reported time spent in the community before reconviction among all participants discharged from a medium-security psychiatric unit to a community forensic service involving intensive case management, compared to participants receiving standard care between 1983 and 2003. During the study, the community forensic service operated several different health care service delivery options, and there were also periods when no service was operating [45]. Participants discharged to the community forensic service were more likely to be on a restrictive order (*p*=0·01) and had a longer duration of admission in the psychiatric unit (*p*<0·001) compared to those receiving standard services [45]. Those in the forensic intervention group spent less time in the community before being reconvicted, with a median time to reconviction of 5 years, compared to a median of 14 years among those receiving standard care (*p*=0·014) [45].

### Mental Health Medication Management

Farabee and Shen [36] conducted a study specifically exploring the interactive effects of antipsychotic and mood stabilizing medication adherence (measured via hair samples) and cocaine use (measured via urine samples) on subsequent criminal justice involvement among parolees. After controlling for age, gender, and ethnicity, medication adherence was not independently associated with a significant reduction in the risk of recidivism. However, after testing the interaction effect of post-release cocaine use and medication adherence and controlling for the same factors as above, medication adherence was associated with 26% reduced odds of returning to custody within 12 months among participants using cocaine, compared to those not using cocaine.

## Discussion

Our synthesis of 13 studies identified in this review suggests utilization of community mental health services after release from jail or prison can improve criminal justice outcomes for people who use illicit drugs and have mental illness. Four studies demonstrated reductions in criminal justice involvement following exposure to standard community mental health services operating within pre-existing service structures, and a further seven studies reported reductions in criminal justice involvement following exposure to a community mental health intervention tailored to people leaving jail or prison. Two studies found an increase in criminal justice involvement following exposure to community mental health services, with these findings occurring among participants with comparatively greater offending risk profiles as part of program participation eligibility. These findings support the need to deliver and expand mental health services for people who use illicit drugs and have mental illness leaving jail or prison. However, variations in mental health service interventions and their integration with other supports, and differences in target populations, make it difficult to determine the features of community mental health service interventions that are most effective and who they are most effective for.

Four of five studies that examined the impact of access to standard community mental health services reported a reduction in criminal justice involvement [34, 38, 39, 41, 44, 46]. Rates of reimprisonment among people who use illicit drugs and have mental illness reflect a failure to address health and social conditions that predispose this group to continue to engage in reoffending [24, 25, 47]. Our findings suggest addressing gaps in service coverage and facilitating acceptable and convenient pathways to access existing community mental health services could play a key role in supporting successful community transition and avoidance of reincarceration. While service access may be driven by a poorly coordinated interface between the criminal justice and mental health systems [29], the costs associated with the development, implementation, and provision of tailored forensic mental services offering intensive case management and support are typically substantial [37, 48]. Therefore, making better use of community mental health services operating within pre-existing service structures may offer a more cost-effective approach to meeting the needs of many people released from jail or prison who use illicit drugs and have mental illness. However, strengthening referral pathways requires investment in service innovation (e.g., sharing of client clinical information between criminal justice systems and community services to enhance continuity of care) and workforce training to support effective treatment and support to people leaving jail or prison [29].

Studies analysing the effectiveness of tailored criminal justice transitional support programs and case management also demonstrated largely positive results; seven of the eight studies in this group reported a reduction in criminal justice involvement following exposure to a tailored community mental health intervention. In these studies, tailored interventions typically aimed to support people with higher risk profiles (e.g., more serious/violent offence histories, serious mental illness diagnosis, or a previous history of homelessness) and include a number of additional support services [40, 43], making it difficult to determine the effectiveness of the specific mental health service components of each. For example, the intervention assessed by Vigilante et al. [43] involved substance use treatment, financial assistance, employment, education, and housing support in combination with mental health services. Similarly, the CORP assessed by Kesten et al. [40] involved intensive group counseling sessions run by a psychologist, along with life skills training, housing, and vocational assistance. There are myriad challenges experienced by people transitioning from prison to the community that require a range of services and support, such as housing, financial support, and accessing health care services [49–51]. Substance use disorder and mental illness are often factors used to identify people at risk of reoffending and who require more intensive support and tailored services, as demonstrated by this systematic review. Research on the effectiveness of social supports such as housing [52] and employment services [53, 54] has shown they are independently effective in reducing criminal justice involvement. Therefore, holistically addressing the needs of individuals leaving jail or prison is likely to be particularly successful in supporting community reintegration, with the effectiveness of tailored mental health and substance use services likely to be augmented when a broader range of services are integrated.

Two studies included in this review found participants receiving community mental health services were more likely to have criminal justice involvement following exposure to such services [35, 45]. However, in both studies, people with exposure to community mental health services had a higher offending risk profile, such as a longer sentence length or a history of more serious criminal offences [35, 45]. These findings suggest the effectiveness of programs in supporting people leaving jail or prison who use illicit drugs and have mental illness must consider varying levels and additional factors that contribute to offending risk. Further, Domino et al. [35] suggested their finding of greater odds of reimprisonment was driven largely by high rates of violations of conditions of release, and that community mental health service contacts may have acted as a catalyst for increased monitoring of conditional release requirements among this group [35]. This illustrates potential unintended harms of mandated community mental health service engagement. While retention in care should be encouraged, mandating participation and penalising those who are non-compliant with criminal sanctions are likely to be counterproductive.

Although our review provides a useful summary of evidence that illustrates the effectiveness of community mental health service contact in reducing future criminal justice involvement among people who use illicit drugs and have mental illness, it has some limitations. All studies were conducted in high-income countries, with 11 of the 13 studies conducted in the USA or Canada. Studies conducted in the USA involved study samples collected from both county jails and state prisons, with only three studies exploring outcomes following release from jails, which limited our ability to analyse differences between the groups. However, given the fundamental differences between these levels of incarceration, consideration should be taken when comparing outcomes among these studies. Only four studies considered gender as a confounder of interest in analysis, with only one study reporting gender as a significant confounder. Due to this, our study did not analyse differences between gender groups. The effectiveness of community mental health services in reducing future criminal justice involvement may differ between countries and in the context of different community mental health service and justice system structures. Nine studies were conducted more than 10 years ago; with deinstitutionalisation and rapid upscaling of community mental health services occurring across many international settings during this time [28, 55, 56], services presented in these studies may have changed. While an extensive list of search terms was used across multiple databases, it is possible more data are available on this research question which we were not able to identify with our search strategy. Additionally, our search strategy was limited to studies in English and within peer-reviewed publications, which may have excluded studies in a language other than English and practice-based evidence that may be published elsewhere. The heterogeneity between studies in the measurement of the primary outcome, such as the proportion of people reoffending versus time spent in the community prior to reimprisonment, precluded the ability to complete a meta-analysis to estimate average impact of exposure to community mental services on criminal justice outcomes. Finally, the inclusion criteria focused on studies of people who use illicit drugs, with definitions of illicit drug use varying substantially across studies, making it difficult to determine the impact of community mental health service contacts according to specific patterns of illicit (e.g., type of illicit drug used, route of administration, frequency of use) or prescription drug use.

This study highlights the paucity of current evidence and the need for updated research exploring the effectiveness of current community mental health services in supporting improved criminal justice outcomes among people who use illicit drugs and have mental illness. To better inform future program investments, studies should explore and compare head-to-head longitudinal criminal justice outcomes following community mental health service contacts based on exposure to pre-existing standard services versus tailored services. Understanding the effectiveness of such services, particularly across varying offender risk profiles and genders, would assist in defining care thresholds and the need to target different population groups. Further, exploration between inpatient and outpatient community mental health services and supports, with outpatient care accounting for a substantial proportion of mental health services, may indicate that upscaling lower-cost outpatient interventions could be effective in reducing criminal justice involvement.

## Conclusion

The substantial impact of cycling in and out of the criminal justice system for people who use illicit drugs and have mental illness demands effective interventions that prevent ongoing criminal justice involvement. This systematic review studied criminal justice outcomes following exposure to community mental health services and tailored interventions after release from jail or prison among people who use illicit drugs and have mental illness. Results support the effectiveness of community mental health service contacts in reducing future criminal justice involvement among these groups. Findings suggest an investment in the strengthening and innovation of community mental health services to meet the needs of people with illicit drug use and mental illness following release from the criminal justice system could deliver significant individual and community benefits. Further longitudinal studies are needed to examine criminal justice outcomes following exposure to community mental health services, with more detailed exploration of the degree of success for specific services within integrated service models to allow for more comprehensive comparison of effectiveness in different settings.

## Supplementary Information

ESM 1 (DOCX 77 kb)

## References

[1] Australian Institute of Health and Welfare. The health of Australia’s prisoners 2018 (2019). Canberra: (AIHW).

[2] Baldry E, McDonnell D, Maplestone P, Peeters M (2016). Ex-prisoners, homelessness and the state in Australia. Aust N Z J Criminol.

[3] Baranyi G, Scholl C, Fazel S, Patel V, Priebe S, Mundt AP (2019). Severe mental illness and substance use disorders in prisoners in low-income and middle-income countries: a systematic review and meta-analysis of prevalence studies. Lancet Glob Health.

[4] Borschmann R, Thomas E, Moran P (2017). Self-harm following release from prison: aA prospective data linkage study. Aust N Z J Psychiatry.

[5] Butler T, Indig D, Allnutt S, Mamoon H (2011). Co-occurring mental illness and substance use disorder among Australian prisoners. Drug Alcohol Rev.

[6] Fazel S, Hayes A, Bartellas K, Clerici M, Trestman R (2016). Mental health of prisoners: pPrevalence, adverse outcomes, and interventions. Lancet Psychiatry.

[7] Kinner SA, Young JT (2018). Understanding and improving the health of people who experience incarceration: an overview and synthesis. Epidemiol Rev.

[8] Fazel S, Yoon IA, Hayes AJ (2017). Substance use disorders in prisoners: an updated systematic review and meta-regression analysis in recently incarcerated men and women. Addiction..

[9] Butler T, Callander D, Simpson M. *National prison entrants’ bloodborne virus and risk behaviour survey report 2004, 2007, 2010, and 2013*. 2015.

[10] Dolan K, Teutsch S, Scheuer N, et al. Incidence and risk for acute hepatitis C infection during imprisonment in Australia. *Eur J Epidemiol*. 2010;25(2):143-148. 10.1007/s10654-009-9421-0. 10.1007/s10654-009-9421-0 20084429

[11] Heard S, Iversen J, Geddes L, Maher L. Australian NSP survey: pPrevalence of HIV, HCV and injecting and sexual behaviour among NSP attendees, 25-year National Data Report 1995-2019. 2020. https://kirby.unsw.edu.au/sites/default/files/kirby/report/ANSPS_25-Year-National-Data-Report-1995-2019.pdf. Accessed 12 Dec 2021

[12] Arum C, Fraser H, Artenie AA, et al. Homelessness, unstable housing, and risk of HIV and hepatitis C virus acquisition among people who inject drugs: a systematic review and meta-analysis. *The Lancet Public Health*. 2021; 10.1016/s2468-2667(21)00013-x. 10.1016/S2468-2667(21)00013-X PMC8097637 33780656

[13] Topp L, Iversen J, Baldry E, Maher L (2013). Collaboration of Australian N. Housing instability among people who inject drugs: results from the Australian needle and syringe program survey. J Urban Health.

[14] Nambiar D, Agius PA, Stoove M, Hickman M, Dietze P. Mortality in the Melbourne injecting drug user cohort study (MIX).(Report). *Harm Reduct J.* 2015;12(1). 10.1186/s12954-015-0089-3. 10.1186/s12954-015-0089-3 PMC4674911 26654430

[15] Degenhardt L, Bucello C, Mathers B (2011). Mortality among regular or dependent users of heroin and other opioids: a systematic review and meta-analysis of cohort studies. Addiction..

[16] Binswanger IA, Blatchford PJ, Mueller SR, Stern MF. Mortality after prison release: opioid overdose and other causes of death, risk factors, and time trends from 1999 to 2009. *Ann Intern Med*. 2013;159(9):592-600. 10.7326/0003-4819-159-9-201311050-00005. 10.7326/0003-4819-159-9-201311050-00005 PMC5242316 24189594

[17] Forsyth SJ, Carroll M, Lennox N, Kinner SA (2018). Incidence and risk factors for mortality after release from prison in Australia: a prospective cohort study. Addiction..

[18] Larney S, Toson B, Burns L, Dolan K (2012). Effect of prison-based opioid substitution treatment and post-release retention in treatment on risk of re-incarceration. Addiction..

[19] Hakansson A, Berglund M. Risk factors for criminal recidivism - a prospective follow-up study in prisoners with substance abuse. *BMC Psychiatry*. 2012;12(1):111. 10.1186/1471-244X-12-111. 10.1186/1471-244X-12-111 PMC3492081 22894706

[20] Butler T, Andrews G, Allnutt S, Sakashita C, Smith NE, Basson J (2006). Mental disorders in Australian prisoners: a comparison with a community sample. Aust N Z J Psychiatry.

[21] Jones D, Maynard A. Suicide in recently released prisoners: a systematic review. *Ment Health Pract*. 2013;17(3):20-27. 10.7748/mhp2013.11.17.3.20.e846.

[22] Al-Rousan T, Rubenstein L, Sieleni B, Deol H, Wallace RB. Inside the nation’'s largest mental health institution: a prevalence study in a state prison system. *BMC Public Health*. 2017;17(1):342. 10.1186/s12889-017-4257-0. 10.1186/s12889-017-4257-0 PMC5397789 28427371

[23] Young JT, Heffernan E, Borschmann R (2018). Dual diagnosis of mental illness and substance use disorder and injury in adults recently released from prison: a prospective cohort study. Lancet Public Health.

[24] Prince JD, Wald C (2018). Risk of criminal justice system involvement among people with co-occurring severe mental illness and substance use disorder. Int J Law Psychiatry.

[25] Blank Wilson A, Draine J, Barrenger S, Hadley T, Evans A (2014). Examining the impact of mental illness and substance use on time till re-incarceration in a county jail. Admin Pol Ment Health.

[26] Gooding P (2016). From deinstitutionalisation to consumer empowerment: mental health policy, neoliberal restructuring and the closure of the ‘'Big bins’' in Victoria. Health Sociol Rev.

[27] Lesage AD, Morissette R, Fortier L, Reinharz D, Contandriopoulos AP (2000). Downsizing psychiatric hospitals: needs for care and services of current and discharged long-stay inpatients. Can J Psychiatr.

[28] Haug H-J, Rössler W (1999). Deinstitutionalization of psychiatric patients in central Europe. Eur Arch Psychiatry Clin Neurosci.

[29] State of Victoria. Royal Commission into Victoria’s Mental Health System, Final Report, Summary, Plain Language Version (2021).Melbourne: (Victorian State Government ). 1-46 https://finalreport.rcvmhs.vic.gov.au/wp-content/uploads/2021/02/RCVMHS_FinalReport_Summary_PlainLanguage.pdf. Accessed 12 Dec 2021

[30] Herbert CW, Morenoff JD, Harding DJ (2015). Homelessness and housing insecurity among former prisoners. RSF..

[31] World Health Organization. Mental health atlas 2017. 2018:1-72. https://www.who.int/mental_health/evidence/atlas/mental_health_atlas_2017/en/. Accessed 13 Dec 2021

[32] Colledge S, Larney S, Peacock A, et al. Depression, post-traumatic stress disorder, suicidality and self-harm among people who inject drugs: aA systematic review and meta-analysis. *Drug Alcohol Depend*. 2020;207. 10.1016/j.drugalcdep.2019.107793. 10.1016/j.drugalcdep.2019.107793 31874449

[33] Moola S, Munn Z, Tufanaru C, et al. JBI checklist for cohort studies. In: Aromataris E, Munn Z, eds. *JBI manual for evidence synthesis*. JBI; 2020:chap Chapter 7: Systematic reviews of etiology and risk.

[34] Constantine RJ, Robst J, Andel R, Teague G (2012). The impact of mental health services on arrests of offenders with a serious mental illness. Law Hum Behav.

[35] Domino ME, Gertner A, Grabert B, Cuddeback GS, Childers T, Morrissey JP (2019). Do timely mental health services reduce re-incarceration among prison releasees with severe mental illness?. Health Serv Res.

[36] Farabee D, Shen H (2004). Antipsychotic medication adherence, cocaine use, and recidivism among a parolee sample. Behav Sci Law.

[37] Godley SH, Finch M, Dougan L, McDonnell M, McDermeit M, Carey A (2000). Case management for dually diagnosed individuals involved in the criminal justice system. J Subst Abus Treat.

[38] Hall DL, Miraglia RP, Lee LW, Chard-Wierschem D, Sawyer D (2012). Predictors of general and violent recidivism among SMI prisoners returning to communities in New York State. The Journal of the American Academy of Psychiatry and the Law.

[39] Hawthorne WB, Folsom DP, Sommerfeld DH (2012). Incarceration among adults who are in the public mental health system: rates, risk factors, and short-term outcomes. Psychiatr Serv.

[40] Kesten KL, Leavitt-Smith E, Rau DR (2012). Recidivism rates among mentally ill inmates: impact of the Connecticut Offender Reentry Program. J Correct Health Care.

[41] Lovell D, Gagliardi GJ, Peterson PD (2002). Recidivism and use of services among persons with mental illness after release from prison. Psychiatr Serv.

[42] Stewart LA, Farrell-MacDonald S, Feeley S (2017). The impact of a community mental health initiative on outcomes for offenders with a serious mental disorder. Crim Behav Ment Health.

[43] Vigilante KC, Flynn MM, Affleck PC (1999). Reduction in recidivism of incarcerated women through primary care, peer counseling, and discharge planning. J Women's Health.

[44] Wang EA, Lin HJ, Aminawung JA, et al. Propensity-matched study of enhanced primary care on contact with the criminal justice system among individuals recently released from prison to New Haven. *BMJ Open*. 2019;9(5):e028097. 10.1136/bmjopen-2018-028097. 10.1136/bmjopen-2018-028097 PMC6502013 31048315

[45] Sahota S, Davies S, Duggan C, Clarke M (2009). The fate of medium secure patients discharged to generic or specialised services. J Forens Psychiatry Psychol.

[46] Green B, Denton M, Heffernan E, Russell B, Stapleton L, Waterson E (2016). From custody to community: outcomes of community-based support for mentally ill prisoners. Psychiatry Psychol Law.

[47] Barrenger SL, Draine J, Angell B, Herman D (2017). Reincarceration risk among men with mental illnesses leaving prison: a risk environment analysis. Community Ment Health J.

[48] Kennedy HG, Simpson A, Haque Q. Perspective on excellence in forensic mental health services: what we can learn from oncology and other medical services. *Perspect Front Psychiatry*. 2019;10(733). 10.3389/fpsyt.2019.00733. 10.3389/fpsyt.2019.00733 PMC6813277 31681042

[49] Hancock N, Smith-Merry J, McKenzie K (2018). Facilitating people living with severe and persistent mental illness to transition from prison to community: a qualitative exploration of staff experiences. Int J Ment Heal Syst.

[50] Cepeda JA, Vetrova MV, Lyubimova AI, Levina OS, Heimer R, Niccolai LM (2015). Community reentry challenges after release from prison among people who inject drugs in St. Petersburg, Russia. Int J Prison Health.

[51] Johnson JE, Schonbrun YC, Peabody ME (2015). Provider experiences with prison care and aftercare for women with co-occurring mental health and substance use disorders: treatment, resource, and systems integration challenges. J Behav Health Serv Res.

[52] Willis M. *Supported housing for prisoners returning to the community: a review of the literature*. Australian Institute of Criminology. 2018.

[53] Nally J, S. L, Ho T, Knutson K. Post-release recidivism and employment among different types of released offenders: a 5-year follow-up study in the United States. *Int J Crim Justice Sci*. 2014;9(1):16.

[54] Tripodi SJ, Kim JS, Bender K (2010). Is employment associated with reduced recidivism?: the complex relationship between employment and crime. Int J Offender Ther Comp Criminol.

[55] Novella EJ (2008). Theoretical accounts on deinstitutionalization and the reform of mental health services: a critical review. Med Health Care Philos.

[56] Rosen A (2006). The Australian experience of deinstitutionalization: interaction of Australian culture with the development and reform of its mental health services. Acta Psychiatr Scand Suppl.

