# Supplementary material for: Criminal Justice Involvement after Release from Prison following Exposure to Community Mental Health Services among People Who Use Illicit Drugs and Have Mental Illness: a Systematic Review
Source: J Urban Health. 2022 May 2;99(4):635–54. doi: 10.1007/s11524-022-00635-5 (PMC9360359; doi:10.1007/s11524-022-00635-5)
Supplement: Supplementary file 1 — (DOCX 77 kb) [file 11524_2022_635_MOESM1_ESM.docx]

**Appendix**

[Appendix 1: PRISMA Checklist 2](#_Toc82787249)

[Appendix 2: Peer reviewed literature search strategy 4](#_Toc82787250)

[2.1 Medline (OVID) search strategy 4](#_Toc82787251)

[2.2 EMBASE search strategy 7](#_Toc82787252)

[2.3 PsycInfo search strategy 9](#_Toc82787253)

[Appendix 3: Joanna Briggs Institute Critical Appraisal Checklist for Cohort Studies 12](#_Toc82787254)

[Appendix 4: Flowchart 14](#_Toc82787255)

**Appendix 1: PRISMA Checklist**

| **Section and Topic** | **#** | **Checklist item** | **Location where item is reported** |
| --- | --- | --- | --- |
| **TITLE** | | |  |
| Title | 1 | Identify the report as a systematic review. | p1 |
| **ABSTRACT** | | |  |
| Abstract | 2 | See the PRISMA 2020 for Abstracts checklist. | p2 |
| **INTRODUCTION** | | |  |
| Rationale | 3 | Describe the rationale for the review in the context of existing knowledge. | p3–4 |
| Objectives | 4 | Provide an explicit statement of the objective(s) or question(s) the review addresses. | P3–4 |
| **METHODS** | | |  |
| Eligibility criteria | 5 | Specify the inclusion and exclusion criteria for the review and how studies were grouped for the syntheses. | p4–5 |
| Information sources | 6 | Specify all databases, registers, websites, organisations, reference lists and other sources searched or consulted to identify studies. Specify the date when each source was last searched or consulted. | p4–5 |
| Search strategy | 7 | Present the full search strategies for all databases, registers and websites, including any filters and limits used. | Appendix 2 |
| Selection process | 8 | Specify the methods used to decide whether a study met the inclusion criteria of the review, including how many reviewers screened each record and each report retrieved, whether they worked independently, and if applicable, details of automation tools used in the process. | p4–5 |
| Data collection process | 9 | Specify the methods used to collect data from reports, including how many reviewers collected data from each report, whether they worked independently, any processes for obtaining or confirming data from study investigators, and if applicable, details of automation tools used in the process. | p5 |
| Data items | 10a | List and define all outcomes for which data were sought. Specify whether all results that were compatible with each outcome domain in each study were sought (e.g. for all measures, time points, analyses), and if not, the methods used to decide which results to collect. | p5 & Table 1 |
|  | 10b | List and define all other variables for which data were sought (e.g. participant and intervention characteristics, funding sources). Describe any assumptions made about any missing or unclear information. | p5 & Table 1 |
| Study risk of bias assessment | 11 | Specify the methods used to assess risk of bias in the included studies, including details of the tool(s) used, how many reviewers assessed each study and whether they worked independently, and if applicable, details of automation tools used in the process. | Appendix 3 |
| Effect measures | 12 | Specify for each outcome the effect measure(s) (e.g. risk ratio, mean difference) used in the synthesis or presentation of results. | Table 1 |
| Synthesis methods | 13 | Describe the processes used to decide which studies were eligible for each synthesis (e.g. tabulating the study intervention characteristics and comparing against the planned groups for each synthesis (item #5)). | p5 &  Table 1 |
| Reporting bias assessment | 14 | Describe any methods used to assess risk of bias due to missing results in a synthesis (arising from reporting biases). | Appendix 3 |
| Additional analyses | 15 | Describe any methods used to assess certainty (or confidence) in the body of evidence for an outcome. | n/a |
| **RESULTS** | | |  |
| Study selection | 16a | Describe the results of the search and selection process, from the number of records identified in the search to the number of studies included in the review, ideally using a flow diagram. | p5 & Appendix 4 |
|  | 16b | Cite studies that might appear to meet the inclusion criteria, but which were excluded, and explain why they were excluded. | Appendix 4 |
| Study characteristics | 17 | Cite each included study and present its characteristics. | p5–6 & Table 1 |
| Risk of bias in studies | 18 | Present assessments of risk of bias for each included study. | Appendix 3 |
| Results of individual studies | 19 | For all outcomes, present, for each study: (a) summary statistics for each group (where appropriate) and (b) an effect estimate and its precision (e.g. confidence/credible interval), ideally using structured tables or plots. | p5–10 |
| Results of syntheses | 20 | Present results of each meta-analysis done, including confidence intervals and measures of consistency. | n/a |
| Reporting biases | 21 | Present assessments of risk of bias due to missing results (arising from reporting biases) for each synthesis assessed. | Appendix 3 |
| Additional analyses | 22 | Give results of additional analyses, if done (e.g., sensitivity or subgroup analyses, meta-regression [see Item 16]). | n/a |
| **DISCUSSION** | | |  |
| Discussion | 23a | Provide a general interpretation of the results in the context of other evidence. | p10 |
|  | 23b | Discuss any limitations of the evidence included in the review. | p12 |
|  | 23c | Discuss any limitations of the review processes used. | p12 |
|  | 23d | Discuss implications of the results for practice, policy, and future research. | p10–13 |
| **OTHER INFORMATION** | | |  |
| Registration and protocol | 24 | Provide registration information for the review, including register name and registration number, or state that the review was not registered. | p4 |
| Support | 25 | Describe sources of financial or non-financial support for the review, and the role of the funders or sponsors in the review. | p18 |
| Competing interests | 26 | Declare any competing interests of review authors. | p18 |
| Availability of data, code and other materials | 27 | Report which of the following are publicly available and where they can be found: template data collection forms; data extracted from included studies; data used for all analyses; analytic code; any other materials used in the review. | n/a |

**Appendix 2: Peer reviewed literature search strategy**

Three peer reviewed literature databases were searched: Medline, EMBASE, and PsycInfo. The below tables outline the literature search strategies and terminologies used for each of the databases. As each database uses different terminologies and require searching in different way, it was necessary to develop search strategies for each database.

These searches were developed in close consultation with a specialist systematic review librarian with expertise across all the databases searched.

*2.1 Medline (OVID) search strategy*

Searches were conducted on January 10^th^, 2022.

Keywords are un-bolded and ‘MeSH’ (Medical subject heading) terms in **bold.**

| **Topic 1: Prison and criminal justice** | |
| --- | --- |
|  | **prisons/ or concentration camps/** |
|  | **criminals/ or prisoners/ or "prisoners of war"/ or crime/ or recidivism/ or law enforcement/** |
|  | (offend* or parol* or incarcerat* or probation* or felon* or convict* or inmate* or criminal* or detain* or remand* or prison* or reformator* or defendant* or reimprison* or reincarcerat*).mp. |
|  | (jail* or gaol* or prison* or police custody or community correction* or correction* program* or custodial population* or custodial setting* or custodial group* or custodial environment or "in custody").mp. |
|  | ((high or medium or low or maximum or minimum) adj2 security).mp. |
|  | (court* adj2 order).mp. |
|  | (justice* adj2 involve*).mp. |
|  | ((correction* or custod* or "criminal justice" or incarcerat* or offend*) adj population*).mp. |
|  | ((correction* or custod* or court* or detention* or justic* or judicia* or justicia* or legal* or penal) adj (center* or centre* or facilit* or agenc* or system or organi?ation* or institution* or establishment* or service*)).mp. |
| **Topic 2: Substance use** | |
|  | 1 or 2 or 3 or 4 or 5 or 6 or 7 or 8 or 9 |
|  | **drug users/ or substance-related disorders/ or amphetamine-related disorders/ or cocaine-related disorders/ or inhalant abuse/ or marijuana abuse/ or narcotic-related disorders/ or opioid-related disorders/ or heroin dependence/ or morphine dependence/ or opium dependence/ or phencyclidine abuse/ or psychoses, substance-induced/ or substance abuse, intravenous/ or substance abuse, oral/ or illicit drugs/ or crack cocaine/ or drug utilization/ or drug misuse/ or prescription drug misuse/ or prescription drug overuse/ or heroin/ or methamphetamine/** |
|  | (substance-related disorder* or amphetamine-related disorder* or cocaine-related disorder* or drug overdose* or inhalant abuse or abused inhalant* or abused volatile solvent* or abused volatile inhalant* or opioid-related disorder* or phencyclidine abuse).mp. |
|  | ((drug* or substanc* or narcotic* or chemical* or opiat* or heroin* or opioid* or opium* or methamphetamin* or amphetamin* or cocain* or crack* or ice* or codein* or morphin* or methadon* or buprenorphin* or suboxon* or subutex* or oxycodon* or "crystal meth*" or methamphet* or amphet* or speed* or benzodiazepin* or cannabis* or marijuana* or solvent* or inhalant* or multidrug* or "IV drug*") adj3 (abuse* or "use" or usage* or useage* or user* or using or used or addict* or misuse* or dependen*)).mp. |
|  | (inject* adj (narcotic* or chemical* or opiat* or heroin* or opioid* or opium* or methamphetamin* or amphetamin* or cocain* or crack* or ice* or codein* or morphin* or methadon* or buprenorphin* or suboxon* or subutex* or oxycodon* or "crystal meth*" or methamphet* or amphet* or benzodiazepin*)).mp. |
|  | (dependen* adj3 (abuse* or "use*" or addict* or misuse* or disorder)).mp. |
|  | ((illicit or street or illegal or divert*) adj (drug* or substance*)).mp. |
|  | (drug abus* or "drug us*" or drug misus* or drug dependen* or inject* drug* or intravenous drug* or inject* substance* or intravenous substance* or "who inject* drug*" or "who use* drug*" or IDU or PWUD or PWID).mp. |
|  | 11 or 12 or 13 or 14 or 15 or 16 or 17 |
| **Topic 3: Mental illness** | |
|  | **mental disorders/ or anxiety disorders/ or anxiety, separation/ or neurotic disorders/ or obsessive-compulsive disorder/ or panic disorder/ or "bipolar and related disorders"/ or bipolar disorder/ or "disruptive, impulse control, and conduct disorders"/ or dissociative disorders/ or mood disorders/ or depressive disorder/ or depressive disorder, major/ or depressive disorder, treatment-resistant/ or dysthymic disorder/ or seasonal affective disorder/ or neurocognitive disorders/ or cognition disorders/ or auditory perceptual disorders/ or neurodevelopmental disorders/ or "attention deficit and disruptive behavior disorders"/ or attention deficit disorder with hyperactivity/ or conduct disorder/ or autism spectrum disorder/ or asperger syndrome/ or autistic disorder/ or personality disorders/ or antisocial personality disorder/ or borderline personality disorder/ or compulsive personality disorder/ or dependent personality disorder/ or paranoid personality disorder/ or passive-aggressive personality disorder/ or schizoid personality disorder/ or schizotypal personality disorder/ or "schizophrenia spectrum and other psychotic disorders"/ or affective disorders, psychotic/ or capgras syndrome/ or delusional parasitosis/ or morgellons disease/ or paranoid disorders/ or psychotic disorders/ or psychoses, substance-induced/ or schizophrenia/ or schizophrenia, catatonic/ or schizophrenia, disorganized/ or shared paranoid disorder/ or substance-related disorders/ or amphetamine-related disorders/ or cocaine-related disorders/ or narcotic-related disorders/ or opioid-related disorders/ or "trauma and stressor related disorders"/ or adjustment disorders/ or stress disorders, traumatic/ or combat disorders/ or psychological trauma/ or stress disorders, post-traumatic/ or stress disorders, traumatic, acute/** |
|  | **mental health/ or mental processes/ or self-injurious behavior/ or suicide/ or suicidal ideation/ or suicide, attempted/** |
|  | ((mental* or psychiatric or psychological or psychot*) adj3 (illness* or health or disorder* or disturbance*)).mp. |
|  | ((personality or affect* or neurot* or mood* or bipolar or "bipolar affective" or "bipolar mood" or manic or dissociative or character) adj3 (disorder* or state or illness* or disturbance*)).mp. |
|  | (anxiety or bipolar or schizophreni* or psychos?s* or depressi* or delusion* or hallucinat* or co-occurr* or cooccurr* or co-morbid* or comorbid* or dual diagnos*).mp. |
|  | (insanity or "nervous breakdown" or psychiatric).mp. |
|  | 19 or 20 or 21 or 22 or 23 or 24 |
|  | 18 and 25 |
| **Topic 4: Community mental health services** | |
|  | **mental health services/ or community mental health services/ or counseling/ or directive counseling/ or emergency services, psychiatric/ or social work, psychiatric/ or schizophrenic psychology/ or community mental health centers/ or hospitals, psychiatric/** |
|  | ((mental health or psychiatric or mental or mental illness* or psychol*) adj (service* or program* or hospital* or facilit* or counsel*)).mp. |
|  | (counsel* or social work* or mental health clinic* or community mental health or community psychology service* or mental illness service*).mp. |
|  | 27 or 28 or 29 |
|  | (10 and 30 and 18) or (10 and 30 and 26) |
|  | limit 31 to english language |

*2.2 EMBASE search strategy*

Searches were conducted on January 10^th^, 2022.

Keywords are un-bolded and ‘EMTREE’ (EMBASE subject heading) terms in **bold.**

| **Topic 1: Prison and criminal justice** | |
| --- | --- |
|  | prison/ or prisoner/ or "prisoners of war"/ or crime/ or offender/ or detention/ or detention camp/ or "prisoner of war camp"/ or forensic psychiatry/ or forensic psychology/ or criminal behaviour/ or recidivism/ or criminal justice/ or law enforcement/ or parole/ |
|  | (offend* or parol* or incarcerat* or probation* or felon* or convict* or inmate* or criminal* or detain* or remand* or prison* or reformator* or defendant* or reimprison* or reincarcerat*).mp. |
|  | (jail* or gaol* or prison* or police custody or community correction* or correction* program* or custodial population* or custodial setting* or custodial group* or custodial environment or "in custody").mp. |
|  | ((high or medium or low or maximum or minimum) adj2 security).mp. |
|  | (court* adj2 order).mp. |
|  | (justice* adj2 involve*).mp. |
|  | ((correction* or custod* or "criminal justice" or incarcerat* or offend*) adj population*).mp. |
|  | ((correction* or custod* or court* or detention* or justic* or judicia* or justicia* or legal* or penal) adj (center* or centre* or facilit* or agenc* or system or organi?ation* or institution* or establishment* or service*)).mp. |
|  | 1 or 2 or 3 or 4 or 5 or 6 or 7 or 8 |
| **Topic 2: Substance use** | |
|  | "drug use"/ or "cannabis use"/ or drug utilization/ or substance abuse/ or drug dependence/ or addiction/ or heroin dependence/ or illicit drug inhalation/ or illicit drug/ or multiple drug abuse/ or methamphetamine dependence/ or drug abuse/ or drug misuse/ or narcotic dependence/ or morphine dependence/ or opiate addiction/ or cannabis addiction/ or cocaine dependence/ or intravenous drug abuse/ or injection drug user/ or prescription drug misuse/ or phencyclidine dependence/ or morphine addiction/ or amphetamine abuse/ or analgesic agent abuse/ or illicit drug inhalation/ or illicit drug/ or street drug/ |
|  | (substance-related disorder* or amphetamine-related disorder* or cocaine-related disorder* or drug overdose* or inhalant abuse or abused inhalant* or abused volatile solvent* or abused volatile inhalant* or opioid-related disorder* or phencyclidine abuse).mp. |
|  | ((drug* or substanc* or narcotic* or chemical* or opiat* or heroin* or opioid* or opium* or methamphetamin* or amphetamin* or cocain* or crack* or ice* or codein* or morphin* or methadon* or buprenorphin* or suboxon* or subutex* or oxycodon* or "crystal meth*" or methamphet* or amphet* or speed* or benzodiazepin* or cannabis* or marijuana* or solvent* or inhalant* or multidrug* or "IV drug*") adj3 (abuse* or "use" or usage* or useage* or user* or using or used or addict* or misuse* or dependen*)).mp. |
|  | (inject* adj (narcotic* or chemical* or opiat* or heroin* or opioid* or opium* or methamphetamin* or amphetamin* or cocain* or crack* or ice* or codein* or morphin* or methadon* or buprenorphin* or suboxon* or subutex* or oxycodon* or "crystal meth*" or methamphet* or amphet* or benzodiazepin*)).mp. |
|  | (dependen* adj3 (abuse* or "use*" or addict* or misuse* or disorder)).mp. |
|  | ((illicit or street or illegal or divert*) adj (drug* or substance*)).mp. |
|  | (drug abus* or "drug us*" or drug misus* or drug dependen* or inject* drug* or intravenous drug* or inject* substance* or intravenous substance* or "who inject* drug*" or "who use* drug*" or IDU or PWUD or PWID).mp. |
|  | 10 or 11 or 12 or 13 or 14 or 15 or 16 |
| **Topic 3: Mental illness** | |
|  | mental health/ or mental disease/ or mood disorder/ or affective neurosis/ or neurosis/ or affective psychosis/ or psychosis/ or acute psychosis/ or alcohol psychosis/ or brief psychotic disorder/ or delusion/ or delusional disorder/ or schizophrenia spectrum disorder/ or schizoaffective psychosis/ or schizophrenia/ or paranoid schizophrenia/ or paranoid psychosis/ or paranoia/ or paranoid personality disorder/ or personality disorder/ or antisocial personality disorder/ or avoidant personality disorder/ or borderline state/ or character disorder/ or compulsive personality disorder/ or dependent personality disorder/ or Diogenes syndrome/ or histrionic personality disorder/ or narcissism/ or passive aggressive personality disorder/ or psychopathy/ or schizoidism/ |
|  | "mixed mania and depression"/ or schizotypal personality disorder/ or anxiety disorder/ or anxiety/ or anxiety neurosis/ or bipolar depression/ or bipolar disorder/ or depression/ or mania/ or bipolar I disorder/ or bipolar II disorder/ or bipolar mania/ or rapid cycling bipolar disorder/ or dissociative disorder/ or multiple personality/ or depersonalization/ or post traumatic stress disorder/ or thought disorder/ or abnormal thinking/ or cognitive defect/ or thinking impairment/ or bradyphrenia/ or behavior disorder/ or abnormal behavior/ or attention deficit disorder/ or disruptive behavior/ or impulse control disorder/ or oppositional defiant disorder/ or perception disorder/ or psychomotor disorder/ or psychosocial disorder/ or autism/ or asperger syndrome/ or obsessive compulsive disorder/ or disorders of higher cerebral function/ or mental patient/ or depressive psychosis/ or manic depressive psychosis/ or manic psychosis/ or psychiatric diagnosis/ or drug induced psychosis/ or cannabis-induced psychosis/ or cocaine-induced psychosis/ or methamphetamine-induced psychosis/ or suicide/ or suicidal behavior/ or suicidal ideation/ or suicide attempt/ |
|  | (anxiety or bipolar or schizophreni* or psychos?s* or depressi* or delusion* or hallucinat* or co-occurr* or cooccurr* or co-morbid* or comorbid* or dual diagnos*).mp. |
|  | (insanity or "nervous breakdown" or psychiatric).mp. |
|  | ((mental* or psychiatric or psychological or psychot*) adj3 (illness* or health or disorder* or disturbance*)).mp. |
|  | ((personality or affect* or neurot* or mood* or bipolar or "bipolar affective" or "bipolar mood" or manic or dissociative or character) adj3 (disorder* or state or illness* or disturbance*)).mp. |
|  | 18 or 19 or 20 or 21 or 22 or 23 |
|  | 17 and 24 |
| **Topic 4: Community mental health services** | |
|  | mental health service/ or mental health care/ or "psychological and psychiatric procedures"/ or psychiatric department/ or psychiatric emergency service/ or psychiatric intensive care unit/ or psychiatric nursing/ or community psychiatric nursing/ or community mental health service/ or community mental health centre/ or community mental health/ or mental hospital/ or mental day hospital/ or crisis intervention/ or psychiatric department/ or psychiatric emergency/ or psychiatry/ or psychiatric treatment/ or forensic psychiatry/ or psychiatrist/ or psychologist/ or psychology/ or social psychiatry/ or counseling/ or counselor/ |
|  | ((mental health or psychiatric or psychol* or mental or mental illness*) adj (service* or program* or hospital* or facilit* or counsel*)).mp. |
|  | (counsel* or social work* or mental health clinic* or community mental health or community psychology service* or mental illness service*).mp. |
|  | 26 or 27 or 28 |
|  | (9 and 17 and 29) or (9 and 25 and 29) |
|  | limit 30 to english language |

*2.3 PsycInfo search strategy*

Searches were conducted on January 10^th^, 2022.

Keywords are un-bolded and American Psychological Association index terms (PsycInfo subject heading) terms in **bold.**

| **Topic 1: Prison and criminal justice** | |
| --- | --- |
| **1** | **prisoners/ or "prisoners of war"/ or criminal offenders/ or perpetrators/ or female criminal offenders/ or male criminal offenders/ or mentally ill offenders/ or crime/ or defendants/ or criminal behavior/ or criminal conviction/ or criminal record/ or recidivism/ or incarceration/ or correctional institutions/ or reformatories/ or maximum security facilities/ or criminal justice/ or legal arrest/ or law enforcement/ or parole/ or probation/ or serial crime/ or legal detention/** |
| 2 | (offend* or parol* or incarcerat* or probation* or felon* or convict* or inmate* or criminal* or detain* or remand* or prison* or reformator* or defendant* or reimprison* or reincarcerat*).mp. |
| 3 | (jail* or gaol* or prison* or police custody or community correction* or correction* program* or custodial population* or custodial setting* or custodial group* or custodial environment or "in custody").mp. |
| 4 | ((high or medium or low or maximum or minimum) adj2 security).mp. |
| 5 | (court* adj2 order).mp. |
| 6 | (justice* adj2 involve*).mp. |
| 7 | ((correction* or custod* or "criminal justice" or incarcerat* or offend*) adj population*).mp. |
| 8 | ((correction* or custod* or court* or detention* or justic* or judicia* or justicia* or legal* or penal) adj (center* or centre* or facilit* or agenc* or system or organi?ation* or institution* or establishment* or service*)).mp. |
| 9 | 1 or 2 or 3 or 4 or 5 or 6 or 7 or 8 |
| **Topic 2: Substance use** | |
| **10** | **drug abuse/ or inhalant abuse/ or polydrug abuse/ or "substance use disorder"/ or addiction/ or "cannabis use disorder"/ or drug dependency/ or "opioid use disorder"/ or intravenous drug usage/ or drug usage/ or heroin addiction/ or marijuana usage/ or prescription drug misuse/ or drug addiction/ or polydrug abuse/ or "substance abuse and addiction measures"/ or marijuana usage/ or "substance related and addictive disorders"/ or intravenous injections/** |
| 11 | (substance-related disorder* or amphetamine-related disorder* or cocaine-related disorder* or drug overdose* or inhalant abuse or abused inhalant* or abused volatile solvent* or abused volatile inhalant* or opioid-related disorder* or phencyclidine abuse).mp. |
| 12 | ((drug* or substanc* or narcotic* or chemical* or opiat* or heroin* or opioid* or opium* or methamphetamin* or amphetamin* or cocain* or crack* or ice* or codein* or morphin* or methadon* or buprenorphin* or suboxon* or subutex* or oxycodon* or "crystal meth*" or methamphet* or amphet* or speed* or benzodiazepin* or cannabis* or marijuana* or solvent* or inhalant* or multidrug* or "IV drug*") adj3 (abuse* or "use" or usage* or useage* or user* or using or used or addict* or misuse* or dependen*)).mp. |
| 13 | (dependen* adj3 (abuse* or "use*" or addict* or misuse* or disorder)).mp. |
| 14 | (inject* adj (narcotic* or chemical* or opiat* or heroin* or opioid* or opium* or methamphetamin* or amphetamin* or cocain* or crack* or ice* or codein* or morphin* or methadon* or buprenorphin* or suboxon* or subutex* or oxycodon* or "crystal meth*" or methamphet* or amphet* or benzodiazepin*)).mp. |
| 15 | ((illicit or street or illegal or divert*) adj (drug* or substance*)).mp. |
| 16 | (drug abus* or "drug us*" or drug misus* or drug dependen* or inject* drug* or intravenous drug* or inject* substance* or intravenous substance* or "who inject* drug*" or "who use* drug*" or IDU or PWUD or PWID).mp. |
| 17 | 10 or 11 or 12 or 13 or 14 or 15 or 16 |
| **Topic 3: Mental illness** | |
| **18** | **mental health/ or mental status/ or mental disorders/ or affective disorders/ or anxiety disorders/ or autism spectrum disorders/ or bipolar disorder/ or borderline states/ or chronic mental illness/ or dissociative disorders/ or neurocognitive disorders/ or neurodevelopmental disorders/ or neurosis/ or personality disorders/ or psychosis/ or somatoform disorders/ or "stress and trauma related disorders"/ or "substance related and addictive disorders"/ or thought disturbances/ or abnormal psychology/ or adaptive behavior/ or attention deficit disorder/ or attention deficit disorder with hyperactivity/ or behavior disorders/ or emotional adjustment/ or emotional disturbances/ or homeless mentally ill/ or mentally ill offenders/ or perceptual disturbances/ or personality processes/ or psychiatric symptoms/ or psychodiagnosis/ or psychiatric patients/ or affective disorders/ or disruptive mood dysregulation disorder/ or major depression/ or affective psychosis/ or schizoaffective disorder/ or anxiety disorders/ or generalized anxiety disorder/ or obsessive compulsive disorder/ or panic attack/ or panic disorder/ or separation anxiety disorder/ or anxiety/ or personality disorders/ or antisocial personality disorder/ or avoidant personality disorder/ or borderline personality disorder/ or dependent personality disorder/ or histrionic personality disorder/ or narcissistic personality disorder/ or obsessive compulsive personality disorder/ or paranoid personality disorder/ or passive aggressive personality disorder/ or sadomasochistic personality/ or schizoid personality disorder/ or schizotypal personality disorder/ or disruptive behavior disorders/ or conduct disorder/ or oppositional defiant disorder/ or "emotional and behavioral disorders"/ or bipolar i disorder/ or bipolar ii disorder/ or cyclothymic disorder/ or mania/ or chronic psychosis/ or consciousness disorders/ or delirium/ or acute psychosis/ or acute schizophrenia/ or hallucinosis/ or reactive psychosis/ or schizophrenia/ or catatonic schizophrenia/ or paranoid schizophrenia/ or process schizophrenia/ or "schizophrenia (disorganized type)"/ or schizophreniform disorder/ or undifferentiated schizophrenia/ or acute stress disorder/ or posttraumatic stress disorder/ or complex ptsd/ or adjustment disorders/ or attachment disorders/ or disinhibited social engagement disorder/ or "substance related and addictive disorders"/ or "substance use disorder"/ or confabulation/ or delusions/ or self-destructive behavior/ or self-injurious behavior/ or suicide/ or attempted suicide/ or suicidal ideation/ or suicidality/ or mental disorders due to general medical conditions/** |
| 19 | (insanity or "nervous breakdown" or psychiatric).mp. |
| 20 | ((mental* or psychiatric or psychological or psychot*) adj3 (illness* or health or disorder* or disturbance*)).mp. |
| 21 | ((personality or affect* or neurot* or mood* or bipolar or "bipolar affective" or "bipolar mood" or manic or dissociative or character) adj3 (disorder* or state or illness* or disturbance*)).mp. |
| 22 | (anxiety or bipolar or schizophreni* or psychos?s* or depressi* or delusion* or hallucinat* or co-occurr* or cooccurr* or co-morbid* or comorbid* or dual diagnos*).mp. |
| 23 | 18 or 19 or 20 or 21 or 22 |
| 24 | 17 and 23 |
| **Topic 4: Community mental health services** | |
| **25** | **community mental health/ or assertive community treatment/ or community mental health centers/ or community mental health services/ or community psychiatry/ or community psychology/ or mental health programs/ or mental health programs/ or crisis intervention services/ or suicide prevention centers/ or mental health services/ or primary mental health prevention/ or counseling/ or community counseling/ or psychotherapeutic counseling/ or psychiatric hospitals/ or psychiatric units/ or psychiatric hospital admission/ or psychiatric hospital readmission/ or psychiatric hospital programs/ or therapeutic community/ or psychiatric clinics/ or psychiatric hospitalization/ or psychiatric hospital discharge/ or psychotherapy/ or psychologists/ or clinical psychologists/** |
| 26 | ((mental health or psychiatric or mental or mental illness* or psychol*) adj (service* or program* or hospital* or facilit* or counsel*)).mp. |
| 27 | (counsel* or social work* or mental health clinic* or community mental health or community psychology service* or mental illness service*).mp. |
| 28 | 25 or 26 or 27 |
| 29 | (9 and 17 and 28) or (9 and 24 and 28) |
| 30 | limit 29 to english language |

**Appendix 3: Joanna Briggs Institute Critical Appraisal Checklist for Cohort Studies**

Reviewer________________________________________­­­­___Date______________________

Author­­­­­­­­­­­­­­­­________________________________Year_________Record Number_____________

|  | **Yes** | **No** | **Unclear** | **Not applicable** |
| --- | --- | --- | --- | --- |
| 1. Were the two groups similar and recruited from the same population? | □ | □ | □ | □ |
| 1. Were the exposures measured similarly to assign people to both exposed and unexposed groups? | □ | □ | □ | □ |
| 1. Was the exposure measured in a valid and reliable way? | □ | □ | □ | □ |
| 1. Were confounding factors identified? | □ | □ | □ | □ |
| 1. Were strategies to deal with confounding factors stated? | □ | □ | □ | □ |
| 1. Were the groups/participants free of the outcome at the start of the study (or at the moment of exposure)? | □ | □ | □ | □ |
| 1. Were the outcomes measured in a valid and reliable way? | □ | □ | □ | □ |
| 1. Was the follow up time reported and sufficient to be long enough for outcomes to occur? | □ | □ | □ | □ |
| 1. Was follow up complete, and if not, were the reasons to loss to follow up described and explored? | □ | □ | □ | □ |
| 1. Were strategies to address incomplete follow up utilized? | □ | □ | □ | □ |
| 1. Was appropriate statistical analysis used? | □ | □ | □ | □ |

Overall appraisal: Include □ Exclude □ Seek further info □

Comments (Including reason for exclusion)

____________________________________________________________________________________________________________________________________________________________________________________

| **Table 1** Study-level critical appraisal using the Joanna Briggs Institute Critical Appraisal Checklist for Cohort Studies | | | | | | | | | | | |
| --- | --- | --- | --- | --- | --- | --- | --- | --- | --- | --- | --- |
|  | Groups similar and recruited from the same population? | Exposures measured similarly to assign to exposed/ unexposed | Exposure measured in a valid and reliable way? | Confounding factors identified? | Strategies to deal with confounding factors stated? | Groups/participants free of the outcome at the start of the study (or at the moment of exposure)? | Outcomes measured in a valid and reliable way? | Follow up time reported and sufficient to be long enough for outcomes to occur? | Follow up complete, and if not, were the reasons to loss to follow up described and explored? | Strategies to address incomplete follow up utilized? | Appropriate statistical analysis used? |
| Constantine et al (2012)^34^ | Yes | Yes | Yes | Yes | Yes | Yes | Yes | Yes | NA | NA | Yes |
| Domino et al (2019)^35^ | Yes | Yes | Yes | Yes | Yes | Yes | Yes | Yes | NA | NA | Yes |
| Farabee & Shen (2004)^36^ | Yes | Yes | Yes | Yes | Yes | Yes | Yes | Yes | Unsure | Unsure | Yes |
| Godley et al (2000)^37^ | NA | NA | NA | No | No | Yes | Yes | Yes | No | No | Unsure |
| Green et al (2016)^46^ | Yes | Yes | Yes | Yes | Yes | Yes | Yes | Yes | NA | NA | Yes |
| Hall et al (2012)^38^ | NA | NA | Yes | Yes | Yes | Yes | Yes | Yes | NA | NA | Yes |
| Hawthorne et al (2012)^39^ | Yes | Yes | Yes | Yes | Yes | Yes | Yes | Yes | NA | NA | Yes |
| Kesten et al (2012)^40^ | Yes | Yes | Yes | Yes | Yes | Yes | Yes | Yes | NA | NA | Yes |
| Lovell et al (2002)^41^ | Yes | Yes | Yes | Yes | Yes | Yes | Yes | Yes | NA | NA | Unsure |
| Sahota et al (2009)^45^ | Yes | Yes | Yes | Yes | Yes | Yes | Yes | Yes | NA | NA | Yes |
| Stewart et al (2017)^42^ | Yes | Yes | Yes | Yes | Yes | Yes | Yes | Yes | NA | NA | Yes |
| Vigilante et al (1999)^43^ | Yes | Yes | Yes | Yes | Yes | Yes | Yes | Yes | NA | NA | Yes |
| Wang et al (2019)^44^ | Yes | Yes | Yes | Yes | Yes | Yes | Yes | Yes | NA | NA | Yes |

**Appendix 4: Flowchart**

| Identification |  | MEDLINE (OVID)  1946 to January 10, 2022  (n = 1564) | |  | PsycINFO (OVID)  1806 to January 10, 2022  (n = 2513) |  | Embase (OVID)  1947 to January 10, 2022  (n = 4795) | |
| --- | --- | --- | --- | --- | --- | --- | --- | --- |
|  |  |  | |  |  |  |  | |
|  |  |  |  | | | | |  |
| Screening |  |  | Records after duplicates removed  (n= 6954) | | | | |  |
|  |  |  | |  |  |  |  | |
|  |  |  | |  | Records screened  (n= 6945) |  | Records excluded  (n = 6875) | |
|  |  |  | |  |  |  |  | |
| Eligibility |  |  | |  |  |  |  | |
|  |  |  | |  | Full-text articles assessed for eligibility  (n= 79) |  | Full text articles excluded  (n = 66)   - Incorrect exposure - Incorrect outcome - Incorrect population of interest | |
|  |  |  | |  |  |  |  | |
| Included |  |  | |  |  |  |  | |
|  |  |  | |  | Studies included in qualitative synthesis  (n = 13) |  |  | |
